# Attitudes toward artificial sweetener consumption among Czech adults: a cross-sectional survey

**DOI:** 10.3389/fnut.2026.1833565

**Published:** 2026-06-10

**Authors:** Andrea Mathová, Diana Chrpová, Boris Hučko, Lenka Kourimská

**Affiliations:** Department of Nutrition and Dietetics, Czech University of Life Sciences Prague, Prague, Czechia

**Keywords:** artificial sweeteners, consumer attitudes, nutrition, Public Health, sugar consumption

## Abstract

**Background:**

The increasing consumption of caloric sweeteners has been associated with the development of non-communicable diseases, including obesity and type 2 diabetes. Artificial sweeteners may represent a suitable alternative. However, their acceptance among consumers remains limited.

**Objective:**

This study aimed to assess attitudes toward artificial sweetener consumption among the population of the Czech Republic.

**Methods:**

A cross-sectional questionnaire survey entitled “Attitudes of Respondents Toward the Consumption of Artificial Sweeteners” was conducted among 147 participants from the general population. The collected data were statistically analyzed to evaluate respondents' perceptions of artificial sweeteners compared with caloric sweeteners.

**Results:**

Only 12% of respondents considered artificial sweeteners (e.g., aspartame, acesulfame K, sucralose) to be healthier than caloric sweeteners, indicating a persistent preference for high-energy sweeteners such as sucrose, high-fructose corn syrup, and glucose syrup. The majority of respondents preferred sugar-sweetened beverages and reported limited awareness of the types of sweeteners used in foods and beverages.

**Conclusion:**

These findings suggest that misconceptions and negative perceptions of artificial sweeteners remain prevalent among consumers. Increasing public awareness through targeted educational strategies may help improve understanding of the safety and potential benefits of artificial sweeteners and support efforts to reduce the intake of caloric sweeteners.

## 1 Introduction

Caloric sweeteners, such as sucrose, glucose–fructose syrup, and high-fructose corn syrup, are a common component of modern diets. However, their increasing consumption has been associated with several serious health problems, particularly obesity and the growing prevalence of type 2 diabetes mellitus (1). To reduce these risks, the World Health Organization (WHO) recommends limiting the intake of free sugars to < 10% of total daily energy intake.

Artificial sweeteners (AS) are widely used as substitutes for caloric sweeteners in foods and beverages to help reduce total energy intake and support adherence to these dietary recommendations. Due to their minimal or zero caloric content, they are commonly added to products such as soft drinks, confectionery, and flavored dairy products (2).

The use of AS is subject to strict regulatory oversight. In the United States, the Food and Drug Administration (FDA) has classified several artificial sweeteners, including saccharin, aspartame, and sucralose, as generally recognized as safe (GRAS) (3). In the European Union, artificial sweeteners are evaluated by the European Food Safety Authority (EFSA), which establishes an acceptable daily intake (ADI) before their approval for use in food products. Sweeteners such as acesulfame K, aspartame, cyclamate, and sucralose are currently authorized for use within the EU (4).

In addition to their role in reducing caloric intake, the health effects of AS have been the subject of ongoing scientific debate. While randomized controlled trials generally support their use as a tool for weight management and glycaemic control, recent meta-analyses indicate that artificially sweetened beverages do not significantly affect key metabolic risk factors such as body weight, glucose levels, or lipid profile compared to unsweetened alternatives (5).

Conversely, emerging evidence suggests that artificial sweeteners may influence metabolic health through mechanisms independent of energy intake. In particular, several studies have highlighted their potential impact on the gut microbiota, including alterations in microbial composition and function, which may in turn affect glucose tolerance and host metabolism (6).

Furthermore, consumer perceptions of artificial sweeteners are influenced not only by scientific evidence but also by media coverage, personal beliefs, and the increasing preference for “natural” food products. This shift toward clean-label products has been associated with a growing skepticism toward synthetic additives, including artificial sweeteners, despite their regulatory approval (7). Another survey conducted in 2023 reported considerable consumer skepticism, with approximately 31% of respondents actively avoiding products containing artificial sweeteners and more than half reporting concerns about their potential health risks (8). Similar findings have been reported in regional studies conducted in South Africa (9), which indicate that a substantial proportion of consumers question the safety of artificial sweeteners.

However, data on consumer perceptions and attitudes toward artificial sweeteners in the Czech Republic remain limited. Therefore, the aim of this study was to assess the consumption of artificial sweeteners among the Czech population and evaluate respondents' attitudes toward their use in the diet.

## 2 Materials and methods

A questionnaire survey was used to collect the data. The questionnaire, entitled “Artificial sweeteners in human nutrition”, contained 13 closed questions and one open question. The questionnaire was distributed electronically using the online survey tool Survio (Survio s.r.o., Brno, Czech Republic), and the link to the questionnaire was shared via the social network Facebook. The list of questions and responses is shown in Table 1. All sweeteners referenced in the questionnaire are approved for use in food within the EU based on Regulation (EC) No 1333/2008 and Directive 94/35/EC (10).

**Table 1:** List of questions and responses to the answer.

Question	Responses (in %)
1. Gender	Female (91; 62%) Male (56; 38%)
2. Age	18–25 (63; 43%) 26–40 (33; 22%) 41–50 (36; 25%) 51–60 (8; 5%) 60+ (7; 4%)
3. Highest education level	Elementary (2; 1%) Secondary with vocational certificate (11; 8%) Secondary with high school diploma (49; 33%) Higher vocational (7; 5%) University degree (78; 53%)
4. Have you ever bought a drink or food with artificial sweeteners?	Yes (139; 95%) No (8; 5%)
5. How often do you buy drinks or foods with artificial sweeteners?	Less than once a month (48; 33%) Once a week (29; 20%) Once a month (24; 16%) Twice a week (14; 10%) More often (11; 8%) Do not buy at all (21; 14%)
6. Do you prefer drinks sweetened with artificial sweeteners to sugar-sweetened drinks?	No (79; 54%) I don't care (43; 29%) Yes (25; 17%)
7. Do you prefer the taste of beverages with artificial sweeteners or sugar-sweetened beverages?	With sugar (81; 55%) Respondents were unable to assess (35; 24%) Artificial and sugar-sweetened drinks tasted the same (25; 17%) With artificial sweeteners (6; 4%)
8. Which of these products with artificial sweeteners do you consume most often?	Chewing gum (52; 35%) Beverage (42; 29%) Candy (21; 14%) Yogurt (5; 12%) None (4; 8%) Compote and jam (1; 1%) Table sweetener (2; 1%)
9. In your opinion, are foods sweetened with artificial sweeteners healthier than those sweetened with sugar?	No (89; 61%) Respondents were unable to assess (40; 27%) Yes (18; 12%)
10. Are you forced to consume artificial sweeteners for health reasons?	No (144; 98%) Yes (3; 2%)
11. Have you ever had health problems related to the consumption of artificial sweeteners?	No (139; 95%) Yes (8; 5%)
12. Do you focus on the type of sweetener used in your food or drink?	No (125; 85%) Yes (22; 15%)
13. Do you use artificial sweeteners in the kitchen (e.g., for baking or flavoring food)?	No (125; 85%) Yes (22; 15%)

### 2.1 Study population

The survey was completed by a total of 147 respondents from the Czech Republic via a structured online questionnaire. It was conducted using a non-probability convenience sampling method. Participants were recruited through social media, specifically via a public post on the author's personal Facebook profile and subsequent sharing within relevant community groups. This approach was chosen to facilitate accessibility and ensure the inclusion of respondents within the target age group.

The inclusion criteria were defined as: residence in the Czech Republic and age 18+ years. Before beginning the survey, all potential respondents were informed about the study's objectives, the voluntary and anonymous nature of their participation, and how their data would be processed. Written informed consent was obtained electronically from all participants before they could access the questionnaire. All collected responses were screened for completeness and adherence to the inclusion criteria prior to data analysis.

### 2.2 Statistical data analysis

The results of the questionnaire survey were graphically processed using Microsoft Excel software (Microsoft Corp., Redmond, WA, USA). For the statistical evaluation of the data, a one-sample test on the parameter p and Pearson's chi-square test were used in the Statistica 12 program (Statsoft, Inc., Tulsa, OK, USA). The test on parameter p was used in questions 5, 6, 7, and 9 (to determine whether the responses are consistent with our assumptions), and the Pearson's chi-square test was used in questions 4 and 11 (to find out whether the answers depend on gender). The significance level of the test was set at 0.05.

## 3 Results

### 3.1 . Sociodemographic characteristics

The study sample comprised 147 respondents with a predominant representation of females (62%). The age distribution was skewed toward younger adults, with the largest segment aged 18–25 years, followed by the 41–50 age category. Regarding education, more than half of the participants held a university degree. A comprehensive breakdown of the sociodemographic profile is provided in Table 1 (Q1–Q3).

### 3.2 . Consumption patterns and frequency

The vast majority of respondents (95%) reported prior experience with purchasing products containing AS. However, the frequency of these purchases varied considerably; only a minority of the sample consumed products with artificial sweeteners on a regular weekly basis, while the largest portion of respondents reported infrequent purchases (less than once a month). Statistical analysis confirmed that gender did not significantly influence the likelihood of purchasing AS containing products (*p* = 0.48). All p-values of one sample t- test are shown in Table 2.

**Table 2:** Results of statistical testing.

Question	*p*-value of one-sample *t*-test
5.	0.19 ≫ One third of respondents did not buy products with artificial sweeteners every week.
6.	0.17 ≫ More than half of the respondents did not prefer drinks with artificial sweeteners.
7.	0.56 ≫ At least half of the respondents did not prefer the taste of drinks with artificial sweeteners.
9.	0.12 ≫ At least half of the respondents did not consider artificial sweeteners to be healthier.
Question	**Chi-square test** ***p*****-value**
4.	0.48 ≫ It did not depend on gender.
11.	0.48 ≫ It did not depend on gender.

Among the categories of artificial sweeteners containing goods, chewing gum and beverages were identified as the most frequently consumed, whereas traditional products like jams or table sweeteners showed minimal popularity. Furthermore, artificial sweeteners are rarely used as functional ingredients in domestic food preparation, as 85% of respondents reported they do not use them for cooking or baking.

### 3.3 Preferences and taste perception

Consumption of AS sweetened beverages appears to be driven by factors other than taste preference. More than half of the respondents explicitly preferred sugar sweetened beverages over those with AS, citing a superior taste profile. Interestingly, nearly a quarter of the participants were unable to assess, yet only a negligible fraction (4%) expressed a definitive preference for the taste of artificial sweeteners. These findings suggest a general sensory skepticism toward AS sweetened alternatives within the studied population.

### 3.4 Health perceptions and safety

The general attitude toward the healthfulness of AS was predominantly skeptical or negative. Only a small minority (12%) perceived AS sweetened foods as a healthier alternative to sugar, while the majority remained unconvinced or uncertain. Despite this skepticism, reported adverse health effects were extremely rare (5%), with no significant difference observed between genders (*p* = 0.48).

Furthermore, the choice to consume AS is seldom motivated by specific health reasons, and most consumers exhibit a “low-involvement” behavior, as evidenced by the fact that 85% of respondents do not monitor the specific type of sweetener used in the products they purchase.

## 4 Discussion

The results of this study indicate that although the majority of respondents have previously purchased products containing artificial sweeteners, their overall perception of these substances remains rather negative. Most respondents did not consider artificial sweeteners to be healthier than caloric sweeteners and generally preferred sugar-sweetened beverages. These findings suggest that skepticism toward artificial sweeteners remains prevalent among consumers.

Similar attitudes have been reported in studies conducted in other countries. For example, a survey conducted in South Africa reported that a large proportion of respondents perceived artificial sweeteners as harmful to health, while only a small proportion considered them beneficial. Specifically, 61% of respondents expressed concerns about xylitol, while 45% of respondents reported concerns regarding the consumption of aspartame (9). Comparable findings were also observed in the United States, where the number of people who think artificial sweeteners are unsafe has decreased from 73% in 2019 to 63% in 2020, although it remains relatively high (11). Another study confirming people's negative view on the use of artificial sweeteners was also a study by Daher et al. (12), in which only 14.9% of respondents believed in the healthiness of artificial sweeteners, and when asked if artificial sweeteners cause cancer, only 10% answered that they do not. Improving public knowledge about artificial sweeteners may help consumers make more informed dietary decisions that could positively affect their health and quality of life. Previous research has shown that educational interventions focused on artificial sweeteners can increase their acceptance and reduce negative attitudes related to perceived health risks, while also improving awareness of their potential benefits. Providing evidence-based information through reliable health authorities and qualified healthcare professionals is therefore essential for supporting informed consumer choices regarding the use of artificial sweeteners and their possible health effects. Accurate and trustworthy education may ultimately contribute to healthier decision-making and greater consumer confidence (13).

Taste preference may also play an important role in consumer choices. In the present study, the majority of respondents preferred the taste of sugar-sweetened beverages over beverages containing artificial sweeteners. This finding may reflect long-standing taste preferences and the perception of sugar as a more natural component of the diet. Such preferences may contribute to the continued consumption of sugar-sweetened products despite the well-established health risks associated with excessive sugar intake, including obesity, type 2 diabetes, and cardiovascular diseases. On the contrary, in the study of Pielak et al. (14), the number of respondents who consumed products with sweeteners was more than twice as high as our results, namely 39% of respondents.

Differences in consumption patterns between countries may be influenced by several factors, including cultural dietary habits, consumer preferences, and the availability of products containing artificial sweeteners on the market.

In the present study, no significant association was observed between gender and the purchase of products containing artificial sweeteners, suggesting that consumption patterns may not differ substantially between men and women in the studied Czech adult population. However, this finding contrasts with recent evidence from Saudi Arabia (15), which reported that women consume significantly more products with artificial sweeteners than men. Such discrepancies may stem from regional socio-cultural differences or varying degrees of weight-related dietary concerns, as women in many populations tend to be more inclined toward choosing lower-energy alternatives to manage body weight. Conversely, another study (13) reported that women are less likely to purchase products containing artificial sweeteners due to concerns about their potential negative health effects, including cancer. However, the result of this study may be biased as only 16% of the respondents were men.

The vast majority of respondents in the present study did not report experiencing any health problems related to the consumption of artificial sweeteners, suggesting that adverse effects are perceived as relatively uncommon among consumers. Only a small proportion of participants indicated that they consumed artificial sweeteners due to health-related reasons.

These findings are consistent with suggests that while artificial sweeteners are generally considered safe when consumed within recommended limits, findings regarding their gastrointestinal effects are not entirely consistent. Some studies have reported the occurrence of gastrointestinal symptoms. However, these observations often come from research involving individuals with pre-existing gastrointestinal conditions, such as irritable bowel syndrome (IBS) or other digestive disorders (16). For example, a study published in 2024, in which a large proportion of participants were diagnosed with IBS, found that aspartame consumption was associated with abdominal pain. In contrast, other research conducted in a population without such conditions, comparing the administration of 100 mg of aspartame with a placebo, did not observe any significant differences in the incidence of abdominal pain or bloating (17).

These findings indicate that the occurrence of adverse gastrointestinal symptoms may depend largely on individual susceptibility, particularly the presence of underlying gastrointestinal disorders, rather than representing a universal response to artificial sweeteners.

Another important finding of this study is that most respondents did not pay attention to the specific type of sweetener used in foods and beverages. This suggests that many consumers may have limited knowledge about artificial sweeteners and their regulatory approval and safety assessments. Increasing public awareness and providing clear information about the safety and acceptable intake levels of artificial sweeteners may therefore help improve consumer understanding and potentially influence dietary choices.

This study has several limitations that should be considered when interpreting the results. First, the relatively small sample size limits the generalizability of the findings to the entire Czech population. Second, the questionnaire was distributed primarily through social media, which resulted in a higher proportion of younger respondents and individuals with higher education levels. Finally, the study relied on self-reported data, which may be subject to recall bias or social desirability bias.

Despite these limitations, the study provides valuable insights into consumer attitudes toward artificial sweeteners in the Czech Republic, a topic that has been relatively understudied in this population. The findings highlight the persistence of negative perceptions regarding artificial sweeteners and underscore the importance of public education on their safety and potential role in reducing the intake of caloric sweeteners.

## 5 Conclusion

This study provides insights into consumer attitudes toward artificial sweeteners in the Czech Republic. The results suggest that although many respondents have experience with products containing artificial sweeteners, their overall perception of these substances remains rather negative. Only a small proportion of respondents considered artificial sweeteners to be healthier than caloric sweeteners, indicating that skepticism and limited consumer awareness may influence their acceptance.

These findings may reflect a gap between current scientific evidence and public perception; however, scientific understanding of the health effects of artificial sweeteners continues to evolve. In addition, because of the cross-sectional design of this study, no causal relationships can be established between consumer knowledge, perceptions, and consumption behaviors. Educational initiatives could potentially contribute to improving consumer understanding of artificial sweeteners and support informed dietary choices, although their effectiveness would require further investigation. Future research should include larger and more diverse population samples and longitudinal designs to enable broader generalization and a better understanding of changes in consumer attitudes over time.

## Data Availability

The raw data supporting the conclusions of this article will be made available by the authors, without undue reservation.
